# Caffeic acid phenethyl ester improves high-carbohydrate diet utilization by promoting adipocyte hyperplasia in grass carp (*Ctenopharyngodon idellus*)

**DOI:** 10.1016/j.aninu.2025.03.009

**Published:** 2025-05-31

**Authors:** Shanghong Ji, Lei Song, Zhiqi Tian, Mingkui Wei, Hong Ji, Jian Sun

**Affiliations:** College of Animal Science and Technology, Northwest A&F University, Yangling 712100, China

**Keywords:** Caffeic acid phenethyl ester, Adipose tissue remodeling, Protein deposition, Anti-inflammatory, Grass carp

## Abstract

Enhancing the ability of fish to consume a high-carbohydrate diet (HCD) is a key focus of aquaculture research. The propolis extract, caffeic acid phenethyl ester (CAPE) has anti-inflammatory, hepatoprotective, and glycolytic-promoting properties, but its potential to mitigate metabolic disorders in fish fed a HCD remains uncertain. This study investigated the effects of CAPE on the adaptability and utilization of a HCD in the herbivorous grass carp (*Ctenopharyngodon idellus*), focusing on growth performance, tissue and organ health, and nutrient metabolism. A total of 270 grass carp with an initial body weight of 12.69 ± 0.05 g were divided into five groups (Control, HCD, HCD + C200 [200 mg/kg CAPE], HCD + C500 [500 mg/kg CAPE], and HCD + C800 [800 mg/kg CAPE], respectively) with three replicates per group and fed for 8 weeks. Compared with Control group, the HCD reduced the final body weight, weight gain rate, specific growth rate, protein deposition rate, and crude protein in whole body and muscle (*P* < 0.05), and increased the feed conversion ratio, intraperitoneal fat index, and hepatosomatic index of grass carp (*P* < 0.05), whereas the addition of CAPE reduced these adverse effects (*P* < 0.05). Peroxisome proliferator-activated receptor γ (PPARγ) was activated by CAPE in adipose tissue (*P* < 0.05), but not in the hepatopancreas or muscle. These changes resulted in adipocyte hyperplasia (a smaller and more uniform distribution of adipocytes) and decreased immune cell penetration and inflammation. CAPE promoted the lipolysis and fatty acid β-oxidation in the adipose tissue, hepatopancreas, and muscle. CAPE improved glucose uptake and utilization-related gene expression in the hepatopancreas and muscle, alleviated hepatic steatosis, and promoted mammalian target of rapamycin (*mtor*) gene expression in muscle for grass carp on the HCD (*P* = 0.034). The addition of CAPE to the HCD inhibited inflammatory response in the adipose tissue, hepatopancreas, and muscle, and reduced the levels of alanine aminotransferase, aspartate aminotransferase, glucose, lactic dehydrogenase, low-density lipoprotein cholesterol, and triglycerides in the serum (*P* < 0.05). In summary, CAPE altered the pattern of adipose tissue expansion by promoting adipocyte hyperplasia, thereby promoting glucose and lipid metabolism, and ameliorated the adverse effects of a HCD on inflammation and growth performance in grass carp.

## 1 Introduction

Carbohydrates are recognized as the most cost-effective energy source in aquatic feeds, with protein-saving effects and amino acid activation to effectively reduce dietary costs (Wilson, 1994). In aquatic animals, glucose is oxidized and decomposed, and the energy released is used for life activities, with excess energy stored in organs or tissues, such as the liver and muscles, for glycogen synthesis. However, compared with mammals, fish have a “congenital diabetic constitution” (Shiau, 1997), characterized by persistently elevated blood glucose concentrations following glucose consumption, resulting in persistent hyperglycemia similar to type II diabetes in mammals (Polakof et al., 2012). Peripheral insulin resistance is an important factor that contributes to the limited ability of fish to efficiently metabolize glucose, although the exact mechanism remains unclear (Caruso and Sheridan, 2011).

The utilization of carbohydrates is relatively low in omnivorous fish and usually requires 250 to 400 g/kg carbohydrate in the diet, whereas herbivorous fish have a greater ability to utilize carbohydrates and usually require 300 to 500 g/kg dietary carbohydrates for optimal growth (Li et al., 2016a). Exceeding the optimal carbohydrate content in diets not only diminishes palatability and reduces feed intake but also has a detrimental effect on fish growth, physiological functions and nutrient absorption. These changes potentially result in growth retardation, liver enlargement and the overall deterioration of fish health (Chaiyapechara et al., 2003; Pan et al., 2022; Tian et al., 2012). Studies on juvenile tilapia (*Oreochromis niloticus*) (Xie et al., 2017), tambaqui (*Colossoma macropomum*) (Sandre et al., 2017), and olive flounder (*Paralichthys olivaceus*) (Rahman et al., 2016) have indicated that a high-carbohydrate diet (HCD) can be converted into “neutral” fat in the body. Fat can accumulate in the abdominal cavity, intestinal wall, and liver, resulting in fatty liver conditions. Consequently, suitable feed additives should be selected to enhance glucose metabolism, thereby improving the utilization of dietary carbohydrates in farmed fish.

Caffeic acid phenethyl ester (CAPE) is a natural flavonoid and a key component of bee propolis. The CAPE content of propolis varies in different regions, with propolis from temperate regions being rich in CAPE, but lower within propolis from tropical regions. Chinese propolis has the highest CAPE content of 15 to 29 mg/g (Lv et al., 2021). The beneficial effects of CAPE, including its anti-inflammatory, antioxidant, antiviral and antitumor properties, have been investigated (Pandey et al., 2023; Rao et al., 1992; Tolba et al., 2013). Research in mice has shown that CAPE effectively improves hyperglycemia, glucose tolerance, and hyperlipidemia and reduces the levels of pro-inflammatory cytokines (Nie et al., 2017). Furthermore, CAPE treatment has been found to enhance insulin sensitivity in HepG2 cells exposed to high glucose levels (Nie et al., 2017). The addition of CAPE to high-fat diets can improve fatty liver disease, inflammation, and insulin resistance in mice. CAPE intake in obese individuals, as well as in the early stages of obesity, can help prevent and treat metabolic syndrome (Kim et al., 2018). A study on zebrafish reported that CAPE attenuated neomycin-induced hair cell damage and prevented cell apoptosis (Park et al., 2014). The addition of CAPE to aquaculture diets may therefore ameliorate the negative effects of HCDs.

Grass carp (*Ctenopharyngodon idellus*) is an economically-important freshwater fish in China that prefers natural aquatic plants as a feed source. Aquatic plants offer high crude protein and low crude fiber contents and are abundant in carbohydrates. Therefore, the grass carp is an ideal candidate to study glucose utilization in fish. A dietary carbohydrate level of 15% to 33% is optimal for grass carp growth (Cai et al., 2018; Su et al., 2021). Exceeding 40% of dietary carbohydrate inhibits growth, increases mesenteric fat deposition, and negates the protein-saving benefits of carbohydrates (Cai et al., 2018; Su et al., 2021). Prolonged consumption of a HCD in grass carp leads to elevated blood glucose, an increased hepatosomatic index, and metabolic disorders (Pan et al., 2022; Su et al., 2021; Tian et al., 2020; Wang et al., 2017).

Hence, the current study examined whether CAPE could mitigate the negative effects of a HCD on fish. The regulatory effects of CAPE on the growth, metabolism, and inflammation of grass carp subjected to a HCD were investigated to evaluate a potential strategy for enhancing glucose tolerance and utilization in fish.

## 2 Materials and methods

### 2.1 Animal ethics statement

The study was carried out following the Guidelines for the Use and Care of Animals by the Animal Care Committee at Northwest A&F University, China (NWAFU-DK-20220801).

### 2.2 Experimental design and feeding management

The experimental design followed previous research, where the Control group contained 30% carbohydrates, while the HCD group contained 50% carbohydrates (Wei et al., 2024a). Similar to a study reported in mice (Kim et al., 2018), different levels of CAPE (the content of active ingredients is 98%, Shanghai Yuanye Bio-Technology Co., Ltd., Shanghai, China) (0, 200, 500, and 800 mg/kg, respectively) were added to the HCD, and together with the Control group, these formed five experimental diets: Control, HCD, HCD + C200, HCD + C500, and HCD + C800, respectively. Ingredients and nutrient levels of these diets are listed in Table 1. The diets used in the experiments were created according to the established methods described in earlier research (Jin et al., 2018). Briefly, all components were first ground using an 80-mesh sieve, then weighed and mixed thoroughly by hand. Approximately 70% water was incorporated to transform the feed into a dough, which was subsequently processed through a pellet machine (JL00SL01, Beijing Jinglai Construction Machinery Co., Ltd., Beijing, China) to form pellets with a diameter of 2 mm. Finally, the pellets were dried in an oven at 40 °C until the moisture content fell below 10%, after which they were stored at −20 °C for future use. Furthermore, the level of CAPE in the diet was analyzed by the Beijing ZKGX Research Institute of Science and Technology (Chemical Lab). Briefly, the samples were crushed and extracted with methanol, and then filtered and diluted to obtain the test solution. Next, the content of CAPE was analyzed using liquid chromatography-mass spectrometry (Triple Quad 5500, AB Sciex, Toronto, Ontario, Canada). The results are shown in Table 1.

**Table 1:** Ingredients and nutrient levels of experimental diets (dry matter basis).

Item	Diets^1^				
	Control	HCD	HCD + C200	HCD + C500	HCD + C800
**Ingredients, g/kg**					
Casein	330.00	330.00	330.00	330.00	330.00
Gelatin	80.00	80.00	80.00	80.00	80.00
Corn starch	300.00	500.00	500.00	500.00	500.00
Soybean oil	50.00	50.00	50.00	50.00	50.00
Choline chloride	5.00	5.00	5.00	5.00	5.00
Premix^2^	10.00	10.00	10.00	10.00	10.00
Bentonite	10.00	10.00	10.00	10.00	10.00
Microcrystalline cellulose	214.00	14.00	13.80	13.50	13.20
BHT	1.00	1.00	1.00	1.00	1.00
CAPE	0.00	0.00	0.20	0.50	0.80
Total	100.00	100.00	100.00	100.00	100.00
**Nutrient levels**^3^**,%**					
Gross energy, MJ/kg	15.98	19.21	19.18	19.31	19.27
Moisture	10.03	9.96	10.08	10.09	10.02
Crude protein	34.41	34.36	34.38	34.40	34.43
Crude lipid	4.93	4.97	4.92	5.01	4.96
Organic matter	93.37	93.29	93.32	93.36	93.35
**Actual level of CAPE in feed, mg/kg**					
CAPE^4^	0.00	0.00	186.96	480.35	773.46

HCD = high-carbohydrate diet; CAPE = caffeic acid phenethyl ester; BHT = butylated hydroxytoluene.

1 Control = 30% carbohydrates + 0 mg/kg CAPE; HCD, 50% carbohydrates + 0 mg/kg CAPE; HCD + C200 = 50% carbohydrates + 200 mg/kg CAPE; HCD + C500 = 50% carbohydrates + 500 mg/kg CAPE; HCD + C800 = 50% carbohydrates + 800 mg/kg CAPE.

2 The premix provided the following per kilogram of diets: vitamin A 67 IU, vitamin D 16.2 IU, vitamin E 7.4 g, vitamin K_3_ 340 mg, vitamin B_1_ 670 mg, vitamin B_2_ 1000 mg, vitamin B_6_ 800 mg, vitamin B_12_ 1.4 mg, vitamin C 10 g, D-pantothenic acid 2.65 g, folic acid 330 mg, nicotinamide 5.35 g, choline chloride 35 g, biotin 34 mg, inositol 8 g, Fe 14 g, Cu 350 mg, Zn 4 g, Mn 1.4 mg, Mg 10 g, Co 30 mg, I 40 mg, Se 35 mg.

3 Nutrient levels were measured values.

4 The actual measurement content of CAPE in the diets.

All grass carp used in this study were obtained from Ankang Fisheries Experimental and Demonstration Station of Northwest A&F University in Shanxi, China. Before the feeding trial, all fish (12.69 ± 0.05 g) were reared in net cages in continuous-flow tanks to acclimatize the fish to the experimental environment. During this period, all fish were fed with a commercial diet (Tongwei Co., Ltd., Chengdu, Sichuan, China) three times a day (08:30, 12:30, and 16:30) for one week. A total of 270 healthy fish, with an initial weight of 13.69 ± 0.03 g, were evenly distributed among 15 cages with a capacity of 1 m^3^, and the cages placed in the continuous-flow tanks. The diets were distributed randomly to three repeated nets within every cluster, fed three times a day (08:30, 12:30, and 16:30) at a rate of 2% of body weight per day over a span of 8 weeks. Throughout the feeding experiment, the temperature of the water was sustained at a range of 23 to 28 °C, while the levels of dissolved oxygen varied from 7 to 11 mg/L, and the pH was within the range of 7.5 to 8.0. Additionally, concentrations of ammonia nitrogen were kept below 0.05 mg/L.

### 2.3 Sample collection

At completion of the experiment, fish were euthanized through immersion in MS-222 solution at a concentration of 100 mg/L (Sigma, St. Louis, MO, USA). Two fish per cage were obtained and stored at −20 °C for measuring chemical composition testing of the whole fish. In addition, blood samples were taken from three fish, and measurements were taken for the hepatosomatic index (HI) and intraperitoneal fat index (IFI) of six randomly selected fish in each enclosure. Following this, the hepatopancreas, intraperitoneal adipose tissue, and muscle tissues of the experimental fish (seven fish per enclosure) were carefully separated on ice. One portion of each tissue sample was fixed in 4% paraformaldehyde for subsequent histopathological examination, while the remaining portion was stored at −80 °C in a cryovial for further analysis.

### 2.4 Growth performance

Various parameters such as final body weight (FBW), feed intake (FI), weight gain rate (WGR), specific growth rate (SGR), feed conversion ratio (FCR), and protein deposition rate (PDR) were calculated and recorded. The formulas were as follows:

WGR = 100 × [(Final body weight, g) − (Initial body weight, g)]/(Initial body weight, g);

SGR (%/d) = 100 × [ln (Final body weight, g) − ln (Initial body weight, g)]/Days;

PDR (%) = 100 × (Fish protein gain, g)/(Total protein intake, g);

FCR = (Amount of feed given, g)/(Weight gain, g);

IFI = 100 × (Intraperitoneal fat weight, g)/(Final weight, g);

HI = 100 × (Hepatopancreas weight, g)/(Body weight, g).

### 2.5 Chemical analysis

The chemical composition of all samples (*n* = 3) was determined using methods outlined by AOAC (2023). Moisture was determined by drying to constant weight in an oven (method 934.01). Crude protein and crude lipid were determined using the Kjeldahl method (method 954.01) (N × 6.25) and ethyl ether extraction (method 2003.05), respectively. Organic matter content was determined by subtracting the percentage of ash from 100% dry matter. Gross energy was determined using an oxygen bomb calorimeter (1341Calorimeter, Parr Instrument Company, Moline, IL, USA), following the steps described by the method 9831 (ISO, 1998).

### 2.6 Hematoxylin and eosin (H&E) staining

H&E staining procedures were executed at Chengdu Lilai Biotechnology Co., Ltd., (Chengdu, Sichuan, China). Initially, the tissue samples were preserved in a solution of 4% paraformaldehyde. Following fixation, the tissues underwent a dehydration process using alcohol and were then embedded in paraffin. Paraffin sections of the tissues were sliced to a thickness of 4 μm, which were subsequently stained using the H&E before being fixed with neutral gum for stability. Next, the stained tissue sections were visualized and captured using fluorescent microscope (Eclipse Ni–U, Nikon, Tokyo, Japan). For quantitative analysis, the adipocyte area within these tissue sections was measured using the ImageJ software. This detailed analysis involved examining approximately 5000 cells per individual fish, and a total of nine fish were analyzed for each experimental treatment group.

### 2.7 Triglyceride (TG) content

The TG content in tissue samples was analyzed using a triglyceride assay kit (catalog no. E1013, Applygen Technologies Inc., Beijing, China). The procedure entailed the addition of 20 μL of lysis solution to 1 mg of tissue, with subsequent steps including homogenization, incubation at room temperature for a duration of 10 min, heating of the sample at 70 °C for 10 min, centrifugation at 1340 × *g* for 5 min, and collection of the supernatant for TG content analysis. Absorbance readings were taken at 550 nm utilizing a plate reader (catalog no. 260887, Thermo Scientific, Shanghai, China).

### 2.8 Ethynyl-2' –deoxyuridine (EDU) assay

The instructions of the EDU-488 Cell Proliferation Detection Kit (4 mg/kg) (catalog no. ST067, Beyotime Biotechnology, Shanghai, China) were followed for operation. In short, the experimental fish were fed with EDU (4 mg/kg added to the acclimatization diet) for one week before feeding the experimental diets. After eight weeks of feeding the experimental diet, adipose tissue was collected, washed with phosphate-buffered saline (PBS) to remove blood and residual EDU, and paraffin sections were prepared using standard procedures. Subsequently, the sections were eluted with xylene and an ethanol gradient, as well as PBS. The click reaction solution was then applied and incubated at room temperature for 30 min in the dark before washing with PBS. Antibody staining for the membrane protein Caveolin-1 was performed, followed by DNA staining with Hoechst 33342 solution. Section observations were made and images were captured using fluorescent microscope (Eclipse Ni–U, Nikon, Tokyo, Japan).

### 2.9 Oil red O staining

The hepatopancreas were immersed in 4% paraformaldehyde at room temperature for fixation. Subsequently, the tissue was washed twice with deionized water to remove residual polyformaldehyde solution, dehydrated with gradient sucrose solutions (10%, 20%, and 30%) and embed with optimal cutting (OCT) embedding agent. Prepared frozen sections with a thickness of 6 μm and placed them in a 60% isopropanol solution for 5 min. Slices ware placed in Oil Red O staining solution and stained in the dark for 5 to 10 min. Excess staining solution was slightly washed away with 60% isopropanol. The nucleus was stained with hematoxylin staining solution for 1 to 2 min, washed twice with pure water, rapidly differentiated with 1% hydrochloric acid alcohol for 1 to 2 s, and then thoroughly washed twice with distilled water and rinsed with tap water for 10 min. Water was absorbed around the slice and sealed with glycerol gelatin. Finally, the stained tissue sections were visualized and captured using fluorescent microscope (Eclipse Ni–U, Nikon, Tokyo, Japan).

### 2.10 Immunohistochemical staining

Immunohistochemical staining was performed using tissue sections prepared during H&E staining. The adipose tissue samples were stained with an anti-F4/80 antibody (#bsm-34028M, Beijing Bioson Biotechnology Co., Ltd., Beijing, China) to detect the presence of macrophages. Each group was analyzed with a total of nine sections, with three sections per cage, to calculate the density of crown-like structures (CLS). High-resolution images were obtained using an immunostainer (Leica Bond MAX, Leica Microsystems, Wetzlar, Hesse, Germany).

### 2.11 Serum biochemical index

Blood samples of 1 mL were collected from each fish and allowed to clot at room temperature for 60 min. Subsequently, the samples underwent centrifugation at 2000 × *g* for 20 min at 4 °C to separate the serum. The serum was then analyzed for biochemical indicators, including alanine aminotransferase (ALT), aspartate aminotransferase (AST), glucose (GLU), high-density lipoprotein cholesterol (HDL-C), lactic dehydrogenase (LDH), low-density lipoprotein cholesterol (LDL-C), and TG. The serum biochemistry analysis was carried out using an automatic biochemistry analyzer (BS-240VET, Shenzhen Mindray Bio-Medical Electronics Co., Ltd., Shenzhen, Guangdong, China) and the relevant reagents.

### 2.12 Quantitative real-time PCR (qRT-PCR)

RNA extraction from tissues (2 fish/cage) was carried out using the AG RNAex Pro Reagent (catalog no. AG21101, Hunan Aikerui Bioengineering Co., Ltd., Changsha, Hunan, China), following the instructions provided by the manufacturer. Briefly, approximately 50 mg of tissue was taken and mixed with an appropriate amount of RNAex for homogenization. Following this, chloroform was added, thoroughly mixed, and left for 5 min. After centrifugation at 8000 × *g* for 15 min at 4 °C, the supernatant was collected. Isopropyl alcohol was then added, mixed well, and left for 10 min before centrifugation at 8000 × *g* at 4 °C for 10 min. The supernatant was discarded, and 80% ethanol (pre-cooled at −20 °C) was added. Centrifugation at 5000 × *g* for 5 min at 4 °C was done, the supernatant carefully removed, and the pellet dried at room temperature. Subsequently, the isolated RNA (1 μg) was reverse transcribed into complementary DNA (cDNA) with the HiScript II Q RT SuperMix for qPCR (+gDNA wiper) (catalog no. R223–01, Vazyme Biotech Co., Ltd., Nanjing, Jiangsu, China). The cDNA was then utilized for qRT-PCR on the CFX96TM Real-Time PCR Detection System (catalog no. 12016265, Bio-Rad Laboratories, Hercules, CA, USA). The PCR reaction mixture included 10 μL of 2 × ChamQ SYBR qPCR Master Mix (catalog no. Q311–02, Vazyme Biotech Co., Ltd., Nanjing, Jiangsu, China), 7.8 μL of sterilized double-distilled water, 0.6 μL of each primer (forward and reverse), and 1 μL of the synthesized cDNA. The qRT-PCR protocol involved an initial denaturation step at 95 °C for 30 s, followed by 40 cycles of 95 °C for 10 s and 60 °C for 15 s. All analyses were made two technical replicates. Expression levels of the target genes were calculated using the comparative Ct method (2^−ΔΔCt^). Primer sequences are provided in Table 2.

**Table 2:** Nucleotide sequences of the primers for quantitative real-time PCR.

Gene	GenBank No.	Forward (5′ to 3′)	Reverse (5′ to 3′)
*dgat1*	XM_051873340.1	ACGAGACATCCGCGAGTAAA	TGCATTGGACAGAACCAGCA
*dgat2*	XM_051907885.1	CACCTTCCAAGTACCTTCTG	AGATCCCACTCGCCTATT
*atgl*	HQ845211.2	TCGTGCAAGCGTGTATATG	GCTCGTACTGAGGCAAATTA
*cpt1b*	XM_051870481.1	AAGGGACGTTACTTCAAGGTG	TCCGACTTGTCTGCCAAGAT
*glut4*	XM_051898884.1	TCTATTGGGGGCATGGTGTC	GAGTTGAAGGTGGTCTCAT
*gk*	GU065314.1	GAAGAGCGAGGCTGGAAGG	CAGAATGCCCTTATCCAAATCC
*pk*	KP262353.1	TTTCTCCAGACAGCATGACG	GCCTTTGCGACTTCCCAGA
*pdk4*	XM_051873196.1	ATGTCAGGAACAGGCACAACA	TCACAGGATCCACGCCAAAG
*il-1β*	MK942107.1	GCCAAGTAGCCGAATCACAGA	AAGCCCAAGATATGCAGGAGTC
*il-6*	KC535507.1	AGCCAGCTCCAGGTGAGTGAAG	GACGGCTCTGCATGTGTCGATC
*il-8*	JN663841.1	ATGAGTCTTAGAGGTCTGGGT	ACAGTGAGGGCTAGGAGGG
*tnf-α*	HQ696609.1	CGCTGCTGTCTGCTTCAC	CCTGGTCCTGGTTCACTC
*mtor*	JX854449.1	GGTACTGCAGAGAACTGATG	GAAAGTATGAGGCTGGAAGG
*gcn2*	XM_051867768.1	AGAGCAGACCCAAAACGAGA	GGTTCTCCGTCTGTTCGTGA
β-Actin	DQ211096.1	TCCACCTTCCAGCAGATGTGGATT	AGTTTGAGTCGGCGTGAAGTGGTA

### 2.13 Western blot

Total protein was extracted using the Total Protein Extraction Kit (catalog no. C1055, Applygen Technologies Inc, Beijing, China). Following extraction, the concentration of the proteins was analyzed using the BCA Protein Assay Kit (catalog no. PC0020, Soleibao Technology Co., Ltd., Beijing, China), ensuring accurate quantification for subsequent analysis. Once quantified, the proteins from each sample were subjected to sodium dodecyl sulfate polyAcrylamide gel electrophoresis (SDS-PAGE), a technique involving an acrylamide gel to separate proteins based on their molecular weight. These separated proteins were then transferred to a polyvinylidene difluoride (PVDF) membrane. In order to prevent non-specific binding, the membrane was blocked with 5% non-fat milk at room temperature for 2 h. After blocking, the membrane was incubated overnight at 4 °C with the primary antibody for specific binding. Upon completion of the incubation, the membrane was washed three times with Tris-buffered saline with Tween-20 (TBST) and subsequently incubated with the secondary antibody, at room temperature for 2 h. The primary antibodies used were: glyceraldehyde-3-phosphate dehydrogenase (GAPDH) (catalog no. GB11002, Seville Biotechnology Co., Ltd., Wuhan, Hubei, China), peroxisome proliferator-activated receptor γ (PPARγ) (catalog no. C26H12, Cell Signaling Technology, Danvers, MA, USA). The secondary antibody used was: goat anti-rabbit horseradish peroxidase conjugate (catalog no. A21020, Abbkine Scientific Co., Ltd., Wuhan, Hubei, China). Finally, the bands were visualized and quantified using an enhanced chemiluminescence (ECL) luminescent agent (catalog no. 310212, Zeta Life, Atlanta, GA, USA) and Image J software, respectively. GAPDH expression served as the loading control.

### 2.14 Data statistics and analysis

All data were expressed as means and standard error of the mean (SEM). Normality of data was assessed using the Kolmogorov–Smirnov test, and homogeneity of variances was evaluated with Levene's test. If data did not conform to normality, all dates were log transformed in order to fulfill the assumptions for normality and homogeneity of variance which are required for one-way ANOVA tests (SPSS Statistics Version 25.0, International Business Machines Corporation, Armonk, NY, USA). Analysis of the data was performed using one-way ANOVA in SPSS 25.0 after ensuring normality and homogeneity. If significant (*P* < 0.05), means were compared by Tukey's honest significant difference. The mathematical model for analysis of variance in one-way ANOVA experiments is as follows:

$$X_{ij}=\mu +a_{i}+e_{ij}\text{,}$$

where *X*_*ij*_ denoted the experimental results, *μ* denoted the population mean, *a*_*i*_ denoted the *i*-th treatment effect, *e*_*ij*_ denoted the accumulated experimental error. The *e*_*ij*_ were independent of each other and *e*_*ij*_ ∼ (0, σ^2^).

## 3 Results

### 3.1 CAPE improved the growth performance and feed efficiency of grass carp fed HCD

No statistical difference in FI was found among the groups (Table 3; *P* = 0.562). The effect of HCD on grass carp was evidenced by a reduction in FBW, WGR, SGR, PDR (Table 3; *P* < 0.05), and crude protein levels in whole body and muscle (Table 4; *P* < 0.05), accompanied by an increase in FCR (Table 3; *P* < 0.001). The addition of CAPE mitigated these negative effects (Table 3; *P* < 0.05).

**Table 3:** Effect of CAPE on growth performance and biological indices of the grass carp.

Item	Group^1^					SEM	*P-*value
	Control	HCD	HCD + C200	HCD + C500	HCD + C800		
FBW, g	24.56^a^	21.86^b^	24.40^a^	23.88^a^	23.97^a^	0.486	<0.001
FI, g/fish	15.81	15.31	15.32	15.31	15.39	0.097	0.562
WGR, %	79.40^a^	59.88^b^	78.44^a^	74.76^a^	74.47^a^	3.516	<0.001
SGR, %/d	1.03^a^	0.84^b^	1.03^a^	1.00^a^	0.99^a^	0.035	0.001
PDR, %	29.91^a^	19.27^b^	30.05^a^	28.40^a^	29.09^a^	2.040	<0.001
FCR	1.42^b^	1.88^a^	1.43^b^	1.50^b^	1.50^b^	0.085	<0.001
IFI, %	1.10^c^	1.63^a^	1.43^b^	1.36^b^	1.18^c^	0.093	<0.001
HI, %	3.60^b^	4.27^a^	3.79^b^	3.72^b^	3.51^b^	1.132	0.001

HCD = high-carbohydrate diet; CAPE = caffeic acid phenethyl ester; FBW = final body weight; FI = feed intake; WGR = weight gain rate; SGR = specific growth rate; PDR = protein deposition rate; FCR = feed conversion ratio; IFI = intraperitoneal fat index; HI = hepatosomatic index.

Values in the same row with different superscripts mean significant difference (*P* < 0.05). Values are means and SEM, *n* = 3.

1 Control group = 30% carbohydrates + 0 mg/kg CAPE; HCD, group = 50% carbohydrates + 0 mg/kg CAPE; HCD + C200 group = 50% carbohydrates + 200 mg/kg CAPE; HCD + C500 group = 50% carbohydrates + 500 mg/kg CAPE; HCD + C800 group = 50% carbohydrates + 800 mg/kg CAPE.

**Table 4:** Effect of CAPE on whole body and muscle crude protein content (%) of grass carp.

Item	Group^1^					SEM	*P-*value
	Control	HCD	HCD + C200	HCD + C500	HCD + C800		
Crude protein in whole body	14.63^a^	13.80^b^	14.70^a^	14.64^a^	14.59^a^	0.169	<0.001
Crude protein in muscle	18.29^a^	17.37^b^	18.77^a^	18.79^a^	18.68^a^	0.268	0.013

HCD = high-carbohydrate diet; CAPE = caffeic acid phenethyl ester.

Values in the same row with different superscripts mean significant difference (*P* < 0.05). Values are means and SEM, *n* = 3.

1 Control group = 30% carbohydrates + 0 mg/kg CAPE; HCD, group = 50% carbohydrates + 0 mg/kg CAPE; HCD + C200 group = 50% carbohydrates + 200 mg/kg CAPE; HCD + C500 group = 50% carbohydrates + 500 mg/kg CAPE; HCD + C800 group = 50% carbohydrates + 800 mg/kg CAPE.

### 3.2 Effects of CAPE on the adipose tissue of grass carp fed HCD

**Fig. 1:**
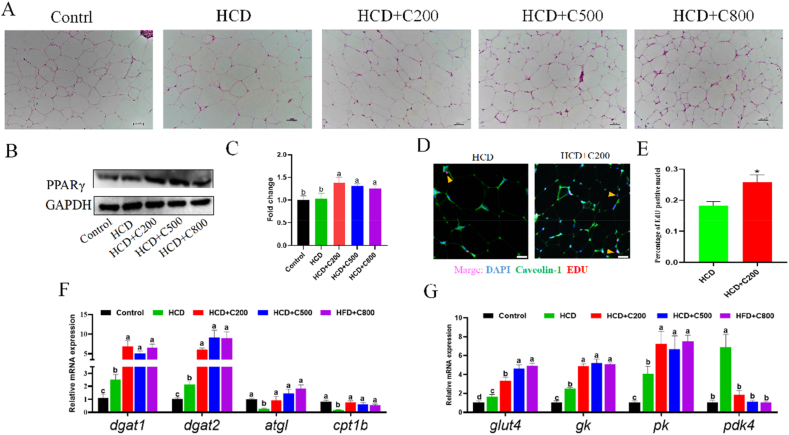
Caffeic acid phenethyl ester (CAPE) promotes adipogenesis and the energy storage and metabolic capacity of adipose tissue in grass carp fed with high-carbohydrate diet (HCD). (A) Hematoxylin and eosin (H&E) stains of the adipose tissue. Magenta: outline of adipocytes; Bluish purple: nucleus. Scar bar = 50 μm. (B) Western blotting of peroxisome proliferator-activated receptor γ (PPARγ) in adipose tissue. (C) Quantification analysis of the protein expression of PPARγ. (D) Ethynyl-2' –deoxyuridine (EDU) stains of adipose tissue. Scar bar = 25 μm. (E) Quantification analysis of EDU positive nucleic. ∗*P* < 0.05. (F) The mRNA expression of *dgat1*, *dgat2, atgl* and *cpt1b* in adipose tissue. (G) The mRNA expression of *glut4*, *gk, pk* and *pdk4* in adipose tissue. GAPDH = glyceraldehyde-3-phosphate dehydrogenase; DAPI = 4′,6-diamidino-2-phenylindole dihydrochloride. Control group = 30% carbohydrates + 0 mg/kg CAPE; HCD group = 50% carbohydrates + 0 mg/kg CAPE; HCD + C200 group = 50% carbohydrates + 200 mg/kg CAPE; HCD + C500 group = 50% carbohydrates + 500 mg/kg CAPE; HCD + C800 group = 50% carbohydrates + 800 mg/kg CAPE. Values with different superscripts mean significant difference (*P* < 0.05). Values are means and SEM, *n* = 3.

H&E staining indicated a reduction in the adipocyte size in the HCD + C groups compared to the HCD group alone (Fig. 1A and Table 5; *P* < 0.001). This was accompanied by a decrease in the IFI (Table 3; *P* < 0.001) and TG contents (Table 6; *P* < 0.001), indicating that CAPE has the potential to reduce lipid accumulation in adipose tissues. Supplementation with CAPE led to an increase in the expression of *PPARγ* (Fig. 1B and C; *P* = 0.037), a key gene involved in regulating adipocyte hyperplasia, and an increase in the proportion of nuclei stained using EdU (Fig. 1D and E; *P* = 0.026). Subsequent analysis indicated that HCD up-regulated TG synthesis-related genes (*dgat1* and *dgat2*) and downregulated lipid metabolism-related genes (*atgl* and *cpt1b*), which was reversed by the CAPE treatment (Fig. 1F; *P* < 0.05). Furthermore, CAPE increased the mRNA levels of glucose metabolism-related genes (*glut4*, *gk,* and *pk*) in adipose tissue, whereas a decrease in the mRNA level of *pdk4* was observed (Fig. 1G; *P* < 0.05). In conclusion, CAPE effectively enhanced glucose and lipid metabolism as well as adipocyte hyperplasia in response to HCD.

**Table 5:** Effect of CAPE on adipocyte size and quantification of crown-like structures (CLS) in the adipose tissue of grass carp.

Item	Group^1^					SEM	*P-*value
	Control	HCD	HCD + C200	HCD + C500	HCD + C800		
Adipocyte size, μm^2^	2572.61^b^	5481.59^a^	2691.25^b^	2629.28^b^	2778.58^b^	563.769	<0.001
CLS, Counts/mm^2^	6.32^b^	23.88^a^	6.64^b^	5.95^b^	5.97^b^	3.534	<0.001

HCD = high-carbohydrate diet; CAPE = caffeic acid phenethyl ester.

Values in the same row with different superscripts mean significant difference (*P* < 0.05). Values are means and SEM, *n* = 3.

1 Control group = 30% carbohydrates + 0 mg/kg CAPE; HCD, group = 50% carbohydrates + 0 mg/kg CAPE; HCD + C200 group = 50% carbohydrates + 200 mg/kg CAPE; HCD + C500 group = 50% carbohydrates + 500 mg/kg CAPE; HCD + C800 group = 50% carbohydrates + 800 mg/kg CAPE.

**Table 6:** Effect of CAPE on triglyceride (TG) content (μmol/g prot) in adipose tissue, hepatopancreas, and muscle of grass carp.

Item	Group^1^					SEM	*P-*value
	Control	HCD	HCD + C200	HCD + C500	HCD + C800		
TG content in adipose tissue	47.62^c^	59.13^a^	54.40^b^	50.71^b^	49.48^c^	2.042	<0.001
TG content in hepatopancreas	6.91^b^	9.55^a^	7.69^b^	7.47^b^	7.50^b^	0.451	0.002
TG content in muscle	1.54^b^	2.06^a^	1.54^b^	1.54^b^	1.68^b^	0.101	0.012

HCD = high-carbohydrate diet; CAPE = caffeic acid phenethyl ester.

Values in the same row with different superscripts mean significant difference (*P* < 0.05). Values are means and SEM, *n* = 3.

1 Control group = 30% carbohydrates + 0 mg/kg CAPE; HCD, group = 50% carbohydrates + 0 mg/kg CAPE; HCD + C200 group = 50% carbohydrates + 200 mg/kg CAPE; HCD + C500 group = 50% carbohydrates + 500 mg/kg CAPE; HCD + C800 group = 50% carbohydrates + 800 mg/kg CAPE.

### 3.3 CAPE relieved hepatic steatosis in grass carp fed HCD

**Fig. 2:**
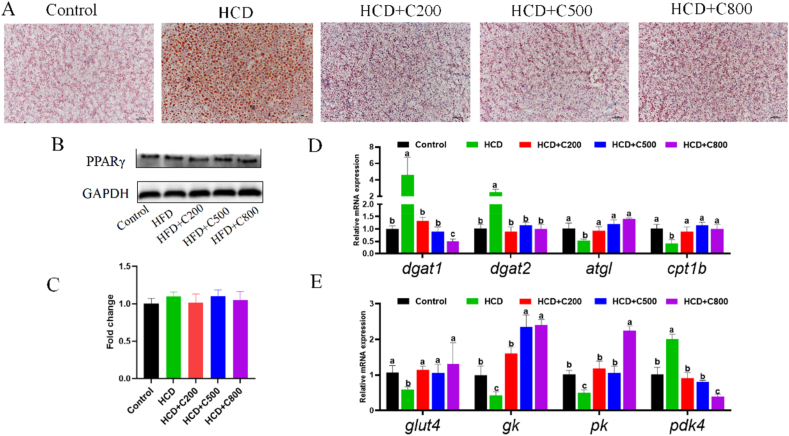
Caffeic acid phenethyl ester (CAPE) promotes glucose utilization and inhibits lipid accumulation in the hepatopancreas of grass carp fed with high-carbohydrate diet (HCD). (A) Oil red O staining of the hepatopancreas. Lipid droplets: orange-red; Nuclei: blue. Scar bar = 50 μm. (B) Western blotting of peroxisome proliferator-activated receptor γ (PPARγ). (C) Quantification of the protein expression of PPARγ. (D) The mRNA expression of *dgat1*, *dgat2, atgl* and *cpt1b* in hepatopancreas. (E) The mRNA expression of *glut4*, *gk, pk* and *pdk4* in hepatopancreas. GAPDH = glyceraldehyde-3-phosphate dehydrogenase. Control group = 30% carbohydrates + 0 mg/kg CAPE; HCD group = 50% carbohydrates + 0 mg/kg CAPE; HCD + C200 group = 50% carbohydrates + 200 mg/kg CAPE; HCD + C500 group = 50% carbohydrates + 500 mg/kg CAPE; HCD + C800 group = 50% carbohydrates + 800 mg/kg CAPE. Values with different superscripts mean significant difference (*P* < 0.05). Values are means and SEM, *n* = 3.

Oil-Red-O staining indicated decreased lipid accumulation in the hepatopancreas of the CAPE-treated grass carp (Fig. 2A). Moreover, the HI and TG contents were reduced in the HCD + C groups (Table 3, Table 6; *P* < 0.05). PPARγ protein expression in the liver differed from that in the adipose tissue and remained relatively stable (Fig. 2B and C; *P* = 0.162). CAPE downregulated the mRNA expression of *dgat1* and *dgat2* and upregulated *atgl* and *cpt1b* (Fig. 2D; *P* < 0.05).

The mRNA levels of glucose metabolism-related genes indicated that CAPE increased the expression of *glut4*, *gk*, *and pk*, while decreasing *pdk4* expression (Fig. 2E; *P* < 0.05). CAPE may therefore prevent excessive lipid accumulation in the hepatopancreas of fish exposed to a HCD, potentially serving as a preventive measure against hepatic steatosis.

### 3.4 CAPE promoted muscle protein deposition in grass carp fed HCD

**Fig. 3:**
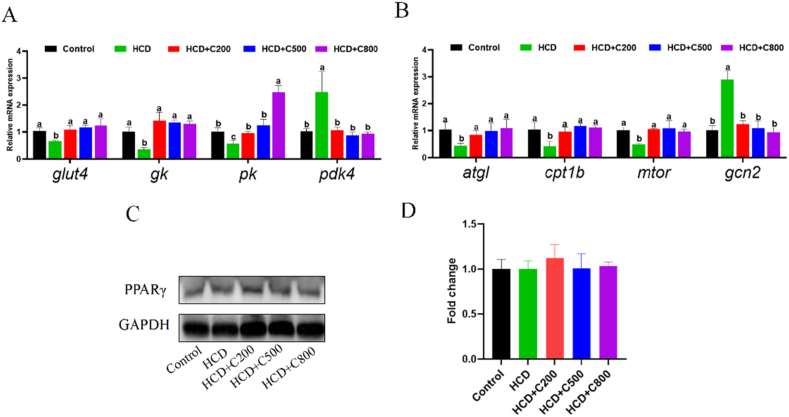
Caffeic acid phenethyl ester (CAPE) promotes glucose and lipid utilization in the muscle of grass carp fed with high-carbohydrate diet (HCD). (A) The mRNA expression of *glut4*, *gk, pk* and *pdk4* in muscle. (B) The mRNA expression of *atgl, cpt1b, mtor* and *gcn2* in muscle. (C) Western blotting of peroxisome proliferator-activated receptor γ (PPARγ). (D) Quantification of the protein expression of PPARγ. GAPDH = glyceraldehyde-3-phosphate dehydrogenase. Control group = 30% carbohydrates + 0 mg/kg CAPE; HCD group = 50% carbohydrates + 0 mg/kg CAPE; HCD + C200 group = 50% carbohydrates + 200 mg/kg CAPE; HCD + C500 group = 50% carbohydrates + 500 mg/kg CAPE; HCD + C800 group = 50% carbohydrates + 800 mg/kg CAPE. Values with different superscripts mean significant difference (*P* < 0.05). Values are means and SEM, *n* = 3.

To assess the effect of adding CAPE to HCD on muscle glucose metabolism, the expression of key genes involved in glucose metabolism was investigated. The HCD + C groups exhibited up-regulated expressions of *glut4*, *gk*, and *pk* compared to the HCD group alone, with a decrease in *pdk4* expression (Fig. 3A; *P* < 0.05). Furthermore, CAPE also increased the mRNA levels of *atgl* and *cpt1* (Fig. 3B; *P* < 0.05), while reducing the TG content (Table 6; *P* = 0.012), indicating an improvement in muscle lipid metabolism.

The protein expression of PPARγ in muscle was similar among the groups (Fig. 3C and D; *P* > 0.05). Elevated mRNA expression of the protein synthesis gene (*mtor*) and decreased expression of the protein degradation gene (*gcn2*) were observed in the HCD + C groups (Fig. 3B; *P* < 0.05). Considering the increase in muscle crude protein content (Table 4; *P* = 0.013), supplementation with CAPE may enhance muscle protein deposition in grass carp fed a HCD.

### 3.5 CAPE alleviated the inflammation in grass carp caused by HCD

**Fig. 4:**
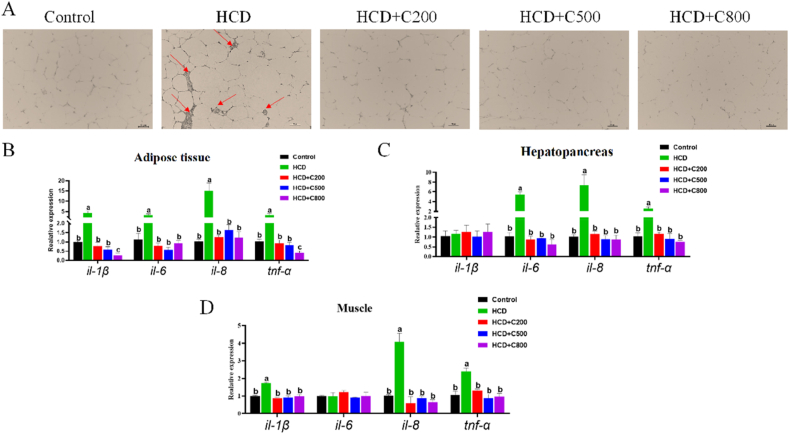
Effects of caffeic acid phenethyl ester (CAPE) on the immunoreaction of grass carp fed with high-carbohydrate diet (HCD). (A) Immunohistochemical staining with an F4/80 antibody of adipose tissue, crown-like structures (CLS) marked by the red arrow. Scar bar = 50 μm. The mRNA expression of *il-1β, il-6, il-8, tnf-α* in adipose tissue (B), hepatopancreas (C) and muscle (D). Control group = 30% carbohydrates + 0 mg/kg CAPE; HCD group = 50% carbohydrates + 0 mg/kg CAPE; HCD + C200 group = 50% carbohydrates + 200 mg/kg CAPE; HCD + C500 group = 50% carbohydrates + 500 mg/kg CAPE; HCD + C800 group = 50% carbohydrates + 800 mg/kg CAPE. Values with different superscripts mean significant difference (*P* < 0.05). Values are means and SEM, *n* = 3.

Considering that CAPE can promote glucose and lipid metabolism in the main metabolic organs of grass carp, the potential impact of CAPE on inflammation was investigated. Immunostaining of adipose tissue (F4/80) indicated that CAPE supplementation inhibited HCD-induced CLS, which appears around dying adipocytes and serves as an indicator of intrinsic inflammation (Fig. 4A). Furthermore, the mRNA levels of *il-1β*, *il-6*, *il-8,* and *tnf-α* (inflammation-related genes) in adipose tissue (Fig. 4B; *P* < 0.05), as well as *il-6*, *il-8*, and *tnf-α* in the hepatopancreas (Fig. 4C; *P* < 0.05), and *Il-1β*, *il-8*, and *tnf-α* in muscle (Fig. 4D; *P* < 0.05) were reduced in the HCD + C groups, compared to the HCD group. Additionally, the CAPE treatment markedly decreased the serum levels of ALT, AST, GLU, LDH, LDL-C, and TG (Table 7; *P* < 0.05), suggesting that CAPE could ameliorate the detrimental effects of the HCD on grass carp health.

**Table 7:** Effect of CAPE on serum biochemical index of grass carp.

Item	Group^1^					SEM	*P-*value
	Control	HCD	HCD + C200	HCD + C500	HCD + C800		
ALT, U/L	3.48^b^	5.80^a^	3.57^b^	3.57^b^	3.63^b^	0.448	<0.001
AST, U/L	39.92^c^	61.17^a^	44.57^b^	43.67 ^b^	44.83^b^	3.691	<0.001
GLU, mmol/L	2.65^b^	3.60^a^	2.67^b^	2.58^b^	2.76^b^	0.189	<0.001
HDL-C, mmol/L	3.12	2.66	3.05	3.00	3.11	0.085	0.559
LDH, U/L	401.14^c^	666.00^a^	434.67^bc^	465.00^b^	416.67^bc^	48.503	<0.001
LDL-C, mmol/L	3.45^b^	4.63^a^	3.50^b^	3.62^b^	3.37^b^	0.232	0.001
TG, mmol/L	3.41^b^	5.04^a^	3.58^b^	3.50^b^	3.55^b^	0.307	0.001

HCD = high-carbohydrate diet; CAPE = caffeic acid phenethyl ester; ALT = alanine aminotransferase; AST = aspartate aminotransferase; GLU = glucose; HDL-C = high-density lipoprotein cholesterol; LDH = lactic dehydrogenase; LDL-C = low-density lipoprotein cholesterol; TG = triglyceride.

Values in the same row with different superscripts mean significant difference (*P* < 0.05). Values are means and SEM, *n* = 3.

1 Control group = 30% carbohydrates + 0 mg/kg CAPE; HCD, group = 50% carbohydrates + 0 mg/kg CAPE; HCD + C200 group = 50% carbohydrates + 200 mg/kg CAPE; HCD + C500 group = 50% carbohydrates + 500 mg/kg CAPE; HCD + C800 group = 50% carbohydrates + 800 mg/kg CAPE.

## 4 Discussion

Improving the utilization of dietary carbohydrates in farmed fish is important for the industry. Fatty liver is a typical disease found in fish fed a HCD; hence, the role of the liver in improving the utilization of dietary carbohydrates has attracted attention. In addition to the hepatopancreas, adipose tissue serves as an important site for fat storage in various fish species, such as Nile tilapia (*Oreochromis niloticus*) (He et al., 2015), zebrafish (*Danio rerio*) (Landgraf et al., 2017), and rainbow trout (Liu et al., 2023). In tilapia, fat tends to accumulate in adipose tissue before other metabolic organs, such as the hepatopancreas or muscle (He et al., 2015), suggesting its potential role as a central hub in the regulation of metabolic disorders. Although the excessive accumulation of abdominal adipose tissue is another common disease in fish, relatively little research has been conducted on the role of adipose tissue in improving dietary carbohydrate utilization. The current study emphasizes that adipose tissue is a potential target for improving the negative effects of HCD in fish.

CAPE was found to promote adipocyte hyperplasia and induce healthy remodeling of grass carp adipose tissue, which concurs with results in mice (Kim et al., 2018). Adipose tissue expansion plays a crucial role in determining whether adipose tissue accumulation leads to metabolic disorders (Albakri et al., 2014; Sakers et al., 2022). Adipose tissue expansion, primarily mediated by adipocyte hypertrophy, exceeds the maximum lipid storage capacity and may result in the ectopic storage of excess free fatty acids in the tissue, thereby inducing metabolic disorders (Gray and Vidal-Puig, 2007; Sun et al., 2013). Under conditions of energy surplus, increasing the number of adipocytes through adipocyte hyperplasia (adipogenesis) can support the healthy expansion of adipose tissue and promote systemic metabolic health (Ghaben and Scherer, 2019). The benefits of adipocyte hyperplasia have been confirmed in grass carp fed high-fat diets (Wei et al., 2024b).

In the current study, CAPE effectively reversed the negative effects of the HCD on grass carp health and growth by promoting adipocyte hyperplasia. This indicates that pathological remodeling of adipose tissue plays an essential role in the intolerance of fish to high fat or carbohydrate diets. The activation of adipocyte hyperplasia may alter the pattern of adipose tissue expansion and enhance the tolerance of grass carp to high fat or carbohydrate diets. Therefore, the current results indicate that the pattern of adipose tissue expansion is an important factor affecting the ability of fish to tolerate high fat or carbohydrate diets.

Adipogenesis is a well-orchestrated, multistep process in which adipocyte precursors commit to form preadipocytes, accumulate nutrients as TG, and then differentiate into mature adipocytes (Zhao et al., 2021). During adipogenesis, glucose uptake, glycolysis, and lipogenesis increase in 3T3-L1 adipocytes (Oates and Antoniewicz, 2022). These processes were upregulated in the CAPE groups, indicating that CAPE increased the capacity of adipose tissue to store glucose by promoting glucose absorption, glycolysis and lipogenesis in the grass carp. CAPE reduced excessive accumulation of adipose tissue induced by the HCD. Promoting adipocyte hyperplasia can increase the metabolic rate of adipose tissue (Feldman et al., 2006). The higher expression of genes related to lipolysis and fatty acid oxidation in the CAPE groups further confirmed this assertion. Therefore, the activation of adipocyte hyperplasia by CAPE could improve the ability of grass carp adipose tissue to store glucose and increase the ability of grass carp adipose tissue to metabolize lipids converted from glucose, thereby increasing the glucose-handling capacity of adipose tissue.

In addition to causing the excessive accumulation of adipose tissue, excessive carbohydrates in fish feed can lead to hepatic steatosis (Amoah et al., 2008; Xu et al., 2016, 2022). The current study indicated that CAPE alleviated HCD-induced fatty liver in grass carp, which is similar to the results in mice (Kim et al., 2018). Kim et al. (2018) reported that CAPE prevented fat from entering the blood and accumulating in non-adipose tissue by signaling the storage of excessive dietary energy in adipose tissue. The decreased serum lipid content and expression of TG synthesis-related genes observed in the current study support this assertion.

CAPE was reported to reduce the expression of *pdk4*, an important enzyme that regulates the activity of pyruvate dehydrogenase, thereby regulating the oxidation of glucose (An et al., 2018). The inhibition of *pdk4* promoted glucose utilization and reduced hepatic lipid deposition in Nile tilapia (Luo et al., 2022) and largemouth bass (Jin et al., 2023). In the current study, CAPE alleviated fatty liver disease by increasing glucose oxidation by reducing the expression of *pdk4* in adipose tissue and muscle. The improvement in the glucose-processing capacity of adipose tissue by CAPE therefore improved glucose oxidation in the metabolic organs of grass carp.

In addition to the hepatopancreas, the muscle is a primary metabolic site for carbohydrate utilization. Under elevated blood glucose concentrations, the muscle glycolysis, glycogen synthesis, and lipid synthesis pathways are activated, enhancing the utilization of exogenous glucose. The current study indicated that HCD inhibited glycolysis and lipid breakdown in muscles, resulting in TG accumulation, which aligns with previous research on mirror carp (*Cyprinus carpio*) (Li et al., 2016b). CAPE promoted muscle glucose and lipid metabolism, leading to reduced TG levels, suggesting that CAPE enhanced the utilization of exogenous glucose. Moreover, CAPE increased the expression of protein synthesis-related genes and crude protein content in muscles, which explains why CAPE improved the growth performance of grass carp. The addition of CAPE may be a viable strategy to enhance the “protein-saving effect” of glucose and address current challenges related to limited feed protein resources, cost reduction, and improvements in production efficiency.

Elevated serum ALT and AST levels indicate hepatocyte membrane rupture and liver injury (Mahmoud and Al-Dera, 2016). Substantially elevated serum levels of ALT and AST have been reported in HCD-fed rats, leading to metabolic strain and liver damage (Nofal et al., 2024), consistent with the findings of the current study. CAPE can protect the liver by reducing serum AST and ALT levels (Shao et al., 2020), which was evidenced in the current study. Therefore, combining the reduction of pro-inflammatory factors in metabolic tissues and the decrease in CLS content in adipose tissue indicates that CAPE can alleviate the inflammatory response to HCD in grass carp and enhance the overall health of fish. Armutcu et al. (2015) emphasized the anti-inflammatory and immunomodulatory activities of CAPE, which has also been reported to substantially reduce the mRNA expression levels of inflammatory factors, such as *tnf-α*, *il-2*, *il-6*, and *iNOS* (Naito et al., 2002; Toyoda et al., 2009).

## 5 Conclusion

**Fig. 5:**
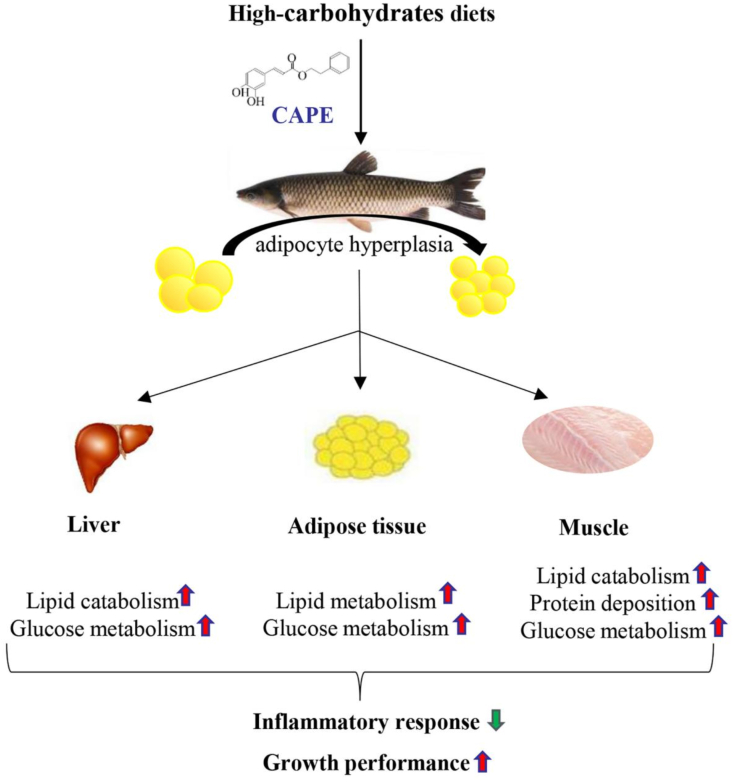
Caffeic acid phenethyl ester (CAPE) can modify the pattern of adipose tissue expansion in response to high carbohydrate intake by promoting adipocyte hyperplasia. This enhancement of glucose and lipid metabolism helps reduce excessive lipid accumulation in the body and promotes muscle protein deposition, ultimately alleviating inflammation and improving growth performance in grass carp on high-carbohydrate diet (HCD).

The current study demonstrated that CAPE could alter the remodeling pattern of adipose tissue by promoting adipocyte hyperplasia. The enhancement of glucose and lipid metabolism reduced excessive lipid accumulation in the body and promoted muscle protein deposition, thereby alleviating fatty liver disease and inflammation, improving growth performance (Fig. 5). Thus, this study offers a comprehensive assessment of the potential applications of CAPE in aquatic diets, presenting potential new solutions to improve glucose tolerance and utilization in farmed fish fed an HCD.

## Credit Author Statement

**Shanghong Ji:** Writing – original draft, Methodology, Formal analysis, Data curation. **Lei Song:** Methodology, Formal analysis, Data curation, Conceptualization. **Zhiqi Tian:** Writing – review & editing. **Mingkui Wei:** Writing – review & editing. **Hong Ji:** Writing – review & editing. **Jian Sun:** Writing – review & editing, Supervision, Resources, Project administration, Funding acquisition.

## Declaration of competing interest

The authors do not have any conflicts of interest to disclose. The paper presents original research and has not been previously submitted or published in any other journal. Each author has made significant contributions to the paper and has consented to its submission to your journal.
