# Supplementary material for: Caffeic acid phenethyl ester improves high-carbohydrate diet utilization by promoting adipocyte hyperplasia in grass carp (Ctenopharyngodon idellus)
Source: Anim Nutr. 2025 May 31;22:154–64. doi: 10.1016/j.aninu.2025.03.009 (PMC12391811; doi:10.1016/j.aninu.2025.03.009)
Supplement: Multimedia component 1 [file mmc1.docx]

**Supplement Table Gene abbreviation and full name.**

| Abbreviations | Full name |
| --- | --- |
| atgl | Adipose triglyceride lipase |
| cpt1b | Carnitine palmitoyl transferase 1b |
| dgat1 | Diacylglycerol acyltransferase 1 |
| dgat2 | Diacylglycerol acyltransferase 2 |
| gcn2 | General control nonderepressible 2 |
| *gk* | Glucokinas |
| *glut4* | Glucose transporter 4 |
| il-1β | Interleukin-1β |
| il-6 | Interleukin-6 |
| il-8 | Interleukin-8 |
| *m*tor | Mammalian target of rapamycin |
| *pdk4* | Pyruvate dehydrogenase kinase 4 |
| *pk* | Pyruvate kinase |
| *pparγ* | Peroxisome proliferator-activated receptor gamma |
| tnf-α | Tumor necrosis factor-α |
